# The prognostic impact of monocyte fluorescence, immunosuppressive monocytes and peripheral blood immune cell numbers in HIV-associated Diffuse Large B-cell Lymphoma

**DOI:** 10.1371/journal.pone.0280044

**Published:** 2023-01-11

**Authors:** Jenifer Vaughan, Tracey Wiggill, Denise Lawrie, Merriam Machaba, Moosa Patel

**Affiliations:** 1 Department of Molecular Medicine and Haematology, Faculty of Health Sciences, University of the Witwatersrand, Johannesburg, South Africa; 2 National Health Laboratory Services, Johannesburg, South Africa; 3 Department of Medicine, Faculty of Health Sciences, University of the Witwatersrand, Johannesburg, South Africa; 4 Clinical Haematology Unit, Chris Hani Baragwanath Academic Hospital, Johannesburg, South Africa; University of Kentucky, UNITED STATES

## Abstract

**Introduction:**

Diffuse large B-cell lymphoma (DLBCL) is a high grade non-Hodgkin lymphoma which is common among immunodeficient people. Derangements of peripheral blood immune cells have been described to have a prognostic impact in DLBCL in high income countries, including a monocytosis, the ratios of lymphocytes to both monocytes (L:M) and neutrophils (N:L), as well as the numbers of regulatory T-cells (Tregs) and immunosuppressive monocytes (HLA-DR_low_ monos). To date, the impact of these variables has not been assessed in the setting of HIV-associated DLBCL (HIV-DLBCL), which is among the most common malignancies seen in people living with HIV. In this study, we assessed these factors in a cohort of South African patients with DLBCL and a high HIV-seropositivity-rate. In addition, we evaluated the prognostic value of monocyte activation (as reflected by monocyte fluorescence (MO-Y) on a Sysmex haematology analyser). This parameter has to date not been assessed in the setting of DLBCL.

**Methods:**

A full blood count and differential count as well as flow cytometry for HLA-DR_low_ monocyte and Treg enumeration were performed in patients with incident DLBCL referred to the Chris Hani Baragwanath Academic Hospital in Johannesburg, South Africa between November 2019 and May 2022. Additional clinical and laboratory data were recorded from the patient charts and laboratory information system.

**Results:**

Seventy-six patients were included, of whom 81.3% were people living with HIV with a median CD4 count of 148 cells/ul. Most patients had advanced stage disease (74.8%) and were predominantly treated with cyclophosphamide, doxorubicin, vincristine, and prednisone (CHOP)-based chemotherapy (without Rituximab). At a median follow-up period of 19 months, the median survival time was 3.5 months, with a 12-month survival rate of 27.0%. All of the immune-cell-related variables (with the exception of the CD4 count) were similar between the people living with HIV and the HIV-negative individuals. In contrast to previous studies, a high monocyte count, the L:M and increased numbers of HLA-DR_low_ monocytes were not significantly associated with survival in HIV-DLBCL, while a neutrophilia (>8 x 10^9^/L), the N:L (>6:1), high numbers of Tregs (≥5.17% of CD4s) and lymphopenia (<1.3 x 10^9^/L) were. In addition, increased monocyte fluorescence (MO-Y >115.5) was associated with superior outcomes, which we speculate to reflect a more robust antitumour immune response among individuals with high levels of monocyte activation. On Cox Proportional hazard analysis, immune-cell factors independently associated with survival included a CD4 count <150 cells/ul and a neutrophilia.

**Conclusion:**

The monocyte count, L:M and the number of HLA-DR_low_ monos are not strong prognostic indicators in HIV-DLBCL, while a low CD4 count and neutrophilia are. Elevation of the MO-Y shows some promise as a potential biomarker of antitumour immunity; further study in this regard would be of interest.

## Introduction

The immune system plays a key role in preventing the evolution of cancer through the recognition and elimination of malignant cells, as well as through clearance of oncogenic viruses. This is evidenced by the increased predisposition to cancer seen among people living with HIV infection and other immunodeficiencies (such as inborn errors of immunity), where chronic immune stimulation due to recurrent or persistent infections predisposes particularly to malignant lymphoid proliferations (such as non-Hodgkin lymphoma (NHL)).

Monocytes and neutrophils are peripheral blood immune cells which play a key role in the innate immune response. Monocytes are produced in the bone marrow, and circulate for 1–3 days in the peripheral blood before egressing into tissues where they give rise to macrophages and dendritic cells. Monocytes play a role in both normal and malignant B- and T-cell function, and can support proliferation of malignant lymphoid cells [1]. Moreover, skewed monocyte differentiation induced by the production of immune mediators (such as IL-10) can dampen the anti-cancer immune response [2]. Neutrophils are short-lived cells which play a critical role in anti-bacterial defense. In addition, they have been shown to produce cytokines which support B-cell survival and differentiation into plasma cells, including B cell-activating factor (BAFF) and a proliferation-inducing ligand (APRIL) [3]. Diffuse large B-cell Lymphoma (DLBCL) is a form of high grade NHL which is particularly common among people with immunodeficiency. It is typically seen among older individuals in high income countries (likely due to a degree of immune senescence), whereas in Africa it is most commonly seen among people living with HIV. The peripheral blood monocyte count [4–6] and the ratios of lymphocytes to both monocytes (L:M) [7, 8] and neutrophils (N:L) [9, 10] in the peripheral blood have been previously shown to have prognostic impact in DLBCL in studies from high income countries, as have the numbers of regulatory T-cells (Tregs) and monocytes with low expression of HLA-DR (HLA-DR_low_ monos) [11] (also known as immunosuppressive monocytes). Both Tregs and HLA-DR_low_ monos form part of the homeostatic response to immune activation, functioning to downregulate the immune response and prevent immune-mediated tissue damage. To date, the impact of the monocyte count, the L:M, the N:L and the number of both Tregs and HLA-DR_low_ monos have not been assessed in the setting of HIV-associated DLBCL (HIV-DLBCL). In this study, we aimed to assess these factors in a cohort of South African patients with DLBCL with a high HIV-seropositivity rate. In addition, we evaluated the prognostic value of monocyte (MO-Y) and neutrophil (NE-SFL) fluorescence, which have to date not been assessed in the setting of DLBCL. These parameters are measured with the routine differential count on Sysmex XN-series haematology analysers (Sysmex, Kobe, Japan), and represent a cost-effective marker of monocyte and neutrophil activation, respectively.

## Materials and methods

This study represents a sub-analysis of a larger study entitled “Evaluation of the association between tumour enrichment with M2-macrophages and survival among South African patients with Diffuse Large B-cell Lymphoma”, which included adult patients (≥18 years of age) with incident DLBCL diagnosed at or referred to Chris Hani Baragwanath Academic Hospital (CHBAH) in Soweto, Johannesburg for specialist management between November 2019 and May 2022. CHBAH is a large tertiary state-sector hospital which provides specialist haematology-oncology services to a large population with poor socio-economic status in the Southern Gauteng province (including the south of the city of Johannesburg), as well as the North West Province of South Africa. All patients referred over this period with an available biopsy of sufficient quality for further immunoshistochemical testing were included. When possible, additional testing was performed on peripheral blood samples, including assessment of the MO-Y, NE-SFL and enumeration of Tregs and HLA-DR_low_ monocytes. Where feasible, residual blood submitted for routine blood tests was used. This additional testing was largely omitted if the patient had been commenced on chemotherapy or corticosteroids prior to enrollment, as these drugs are known to influence immune cell numbers. Treg and HLADR_low_ monocyte analysis was performed only in patients who had not been commenced on corticosteroids. Details regarding the numbers of each research test performed and the reasons for case exclusion are summarized in S1 Appendix. The testing performed included a full blood count and differential white cell count (performed on a Sysmex XN-9000 haematology analyser), which includes the MO-Y and NE-SFL extended differential parameters. These measure the level of a fluorescent marker which binds intracellular RNA in the monocytes and neutrophils, respectively (S2 Appendix). As a measure of the level of intracellular RNA, they are thought to be a marker of cell activation [12, 13]. HLA-DR_low_ monocyte and Treg enumeration were assessed using flow cytometry; the flow cytometry methodology is detailed in the S3 Appendix. Treg enumeration included evaluation of CD39 (a marker associated with a more potent immunosuppressive phenotype) and Helios (a transcription factor thought to reflect thymically derived as opposed to peripherally induced Tregs). Other pertinent information was recorded from the laboratory information system ((TrakCare, InterSystems, Cambridge, Massachusetts, United States) and the patient hospital files. This included details of the clinical presentation and stage, the international prognostic index (IPI) score (based on age, Eastern Cooperative Oncology Group (ECOG) performance status (PS), involvement of extranodal sites, stage and the lactate dehydrogenase (LDH) level), HIV-status and information regarding Anti-retroviral therapy (ART) exposure, the CD4-count and HIV viral load (HIVVL), detailed histology and bone marrow (BM) findings, biochemistry results (LDH, albumin, total protein, kidney function, C-reactive protein (CRP)), details regarding treatment, clinical course and outcomes. Not all data was available in all patients owing to lost or incomplete hospital records/work-up. CD4 counts were measured on a Beckman Coulter FC500 MPL/CellMek platform (Beckman Coulter, California, USA). In addition, CD4 counts were also calculated from the Treg analysis, and confirmed to compare very closely to results from the standard method when both were performed. The calculated CD4 counts were used for analysis when a routine CD4 count was not available (predominantly in the HIV negative patients). For calculation of the lymphocyte to monocyte (L:M) and the neutrophil to lymphocyte (N:L) ratios, patients with circulating small sized clonal B-cells which could not be distinguished from normal lymphocytes were excluded, as were all patients with circulating tumour cells from the MO-Y analysis (as tumour cells may be miscounted as monocytes by automated haematology analysers). In addition, such patients were excluded from survival analysis in relation to the lymphocyte count. The cell of origin (COO), with regard to DLBCL, was determined using the Hans algorithm, where all cases expressing CD10 as well as those negative for CD10 but expressing BCL6 without MUM1 were classified as being of Germinal Centre (GC) origin. All CD10 negative cases expressing BCL6 with MUM1, MUM1 without BCL6 or lacking expression of these markers were classified as being of non-GC origin [14]. The study was approved by the Human Research Ethics Committee (HREC) of the University of the Witwatersrand (ref M190709); all participants from whom peripheral blood was collected solely for the purposes of the study gave informed consent. Informed consent was also obtained from all patients on whom testing was performed on residual samples provided they had not demised in the interim.

### Statistical analysis

Continuous data are presented as the median (interquartile range (IQR)) and categorical data as frequencies and percentages. The Mann-Whitney U-test and Fishers exact test were used to compare continuous and ordinal variables of interest respectively, and correlation between variables of interest was assessed using Spearman’s correlation. Correlations were performed on all the results pooled, irrespective of HIV-status. Owing to limited numbers, cases with low (0–1), intermediate (2–3) and high IPI scores (4–5) were pooled for analysis and referred to as the IPI class. Optimal cut-off values for the lymphocyte count, monocyte count, neutrophil count, L:M, N:L, MO-Y, Treg and HLA-DR_low_ monocyte numbers were determined by Receiver Operator Curve (ROC) analysis, with the values demonstrating the best balance between test sensitivity and specificity for predicting patient survival (as evidenced by a high likelihood ratio (LR) with both high sensitivity and high specificity) being selected. Univariate survival analysis was performed using Kaplan-Meier survival estimates, and median survival times were compared using log rank tests. This was performed on the entire cohort as well as among the people living with HIV (so as to obtain a clearer view of determinants of survival in this subgroup). Unfortunately, this sub-analysis could not be meaningfully performed in the HIV negative patients due to the small size of this group. Patients who were clinically stable but lost to follow-up before they were referred to the Clinical Haematology Unit were excluded from the survival analysis, while those who were lost to follow-up after having commenced treatment were included. The one-year overall survival (OS) rate was calculated as the proportion of patients who were still alive 12 months after diagnosis. Living patients who had been followed up for <12 months were excluded from this analysis. Cox proportional-hazards models were used to investigate the association between the survival time at the time of write-up and predictor variables which showed a statistically significant association with survival on Kaplan-Meier analysis. As with univariate analysis, this was performed both on the whole cohort and specifically among the people living with HIV. The assumptions of proportionality were confirmed to have been met by means of Schoenfield analysis of proportionality. Statistical analysis was performed using Prism software, version 5 (GraphPad Software, San Diego, California, United States), at https://statpages.info/prophaz.html (for Cox proportional hazard regression analysis) and at https://acetabulum.dk/cgi-bin/cox (for proportionality testing). Statistical significance was accepted at a two-sided p-value of 0.05.

## Results

**Fig 1:**
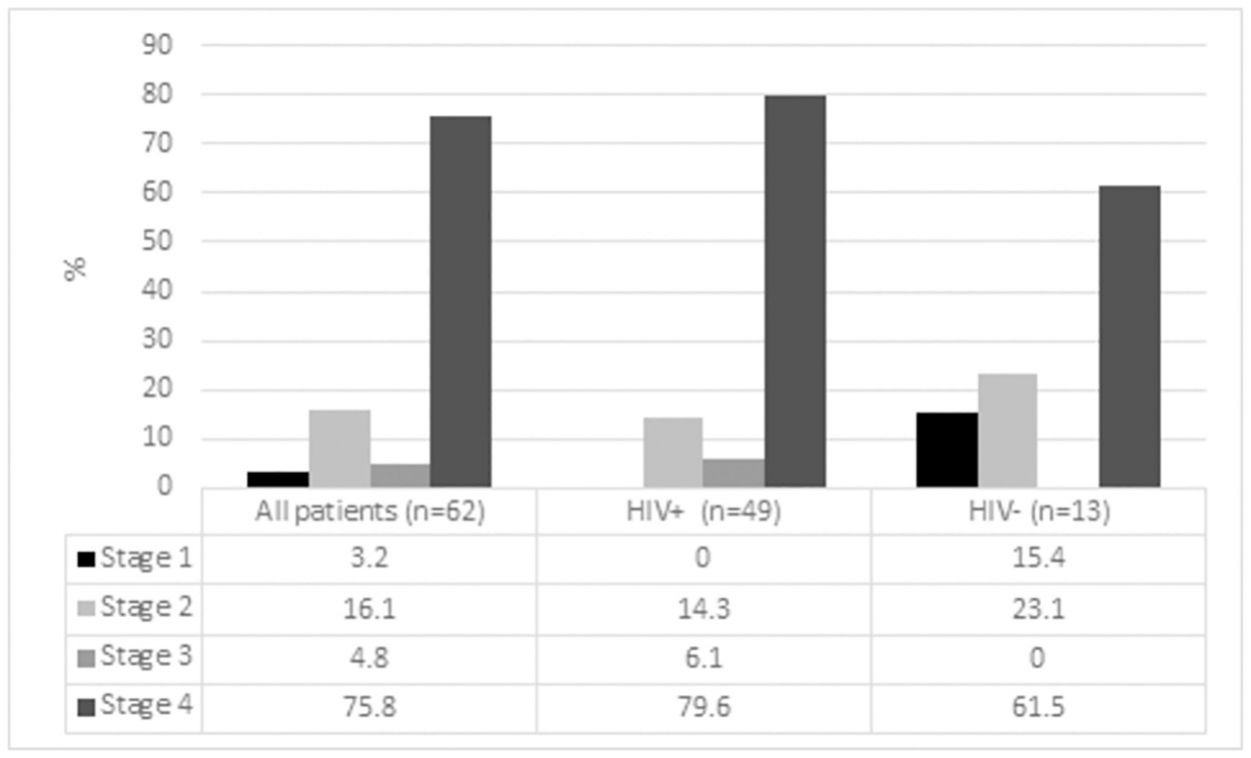
Distribution of disease stage according to HIV status.

**Fig 2:**
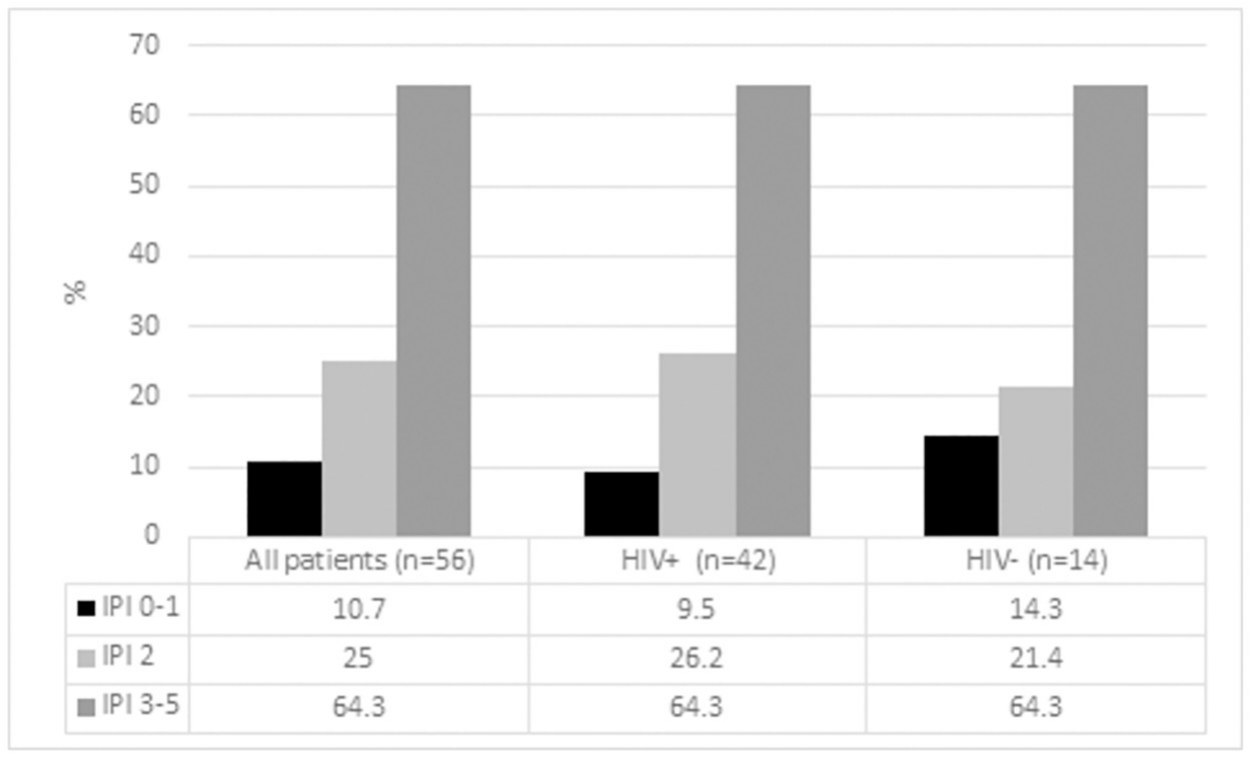
IPI score according to HIV-status.

The study included 76 patients with a median age of 42 years and a male to female ratio of 1.3:1. HIV test results were available in 75/76 of the patients, of whom 61 (81.3%) were people living with HIV. Information about ART exposure was available in 54 patients, 44 of whom (81.5%) were on ART at the time of referral. However, of these, 13 (29.5%) had been commenced on ART after the onset of their symptoms of lymphoma. The CD4 count was <150 cells/ul in 33 (54.1%) of the people living with HIV, of whom 85% were on ART for a median of 6.5 months (IQR 2–45), and 21.4% were virologically suppressed (HIVVL <100 copies/ml). Among patients with higher CD4 counts (>150 cells/ul), virological suppression (VS) was significantly more common (present in 55.6%; p = 0.01), and the duration of ART exposure was significantly longer (24.5 months (IQR 14.3–63); p = 0.048). Relevant clinical and histological data are summarized in Table 1. The majority of patients (irrespective of HIV-status) had advanced stage disease and an intermediate IPI score (Table 1, Figs 1 and 2), while the people living with HIV were significantly younger than their HIV-negative counterparts. Information about chemotherapy exposure was available in 66 patients; chemotherapy was administered in 56 (84.8%) of these; most (44) received cyclophosphamide, doxorubicin, vincristine, and prednisone (CHOP) based treatment as the index chemotherapy protocol (+/- etopiside). Rituximab was used in combination with CHOP in only 8 patients upfront (Table 1), all of whom were HIV negative. Survival data was available in 73 patients; at a median follow-up period of 19 months (IQR 12.3–26 months), the median survival time from diagnosis was 3.5 months, with a 12-month survival rate of 27.0%. Among the patients who demised, 22/50 (44%) died within 1 month of diagnosis, and 9/45 (20%) of those with available information about treatment did not receive any chemotherapy. Among patients who received at least 2 cycles of chemotherapy, the median survival time was 21 months, with a 12-month survival rate of 54.8%. The causes of death are summarized in Table 2.

**Table 1:** Pertinent demographic, HIV-related and other laboratory data.

Parameter	Result	p-value*
Age, median (IQR)	42 (35–54)	
HIV+	41 (33.5–49)	0.02
HIV-	60 (34.8–63.3)	
Male:Female	1.3:1	
HIV+	1.2:1	0.56
HIV-	1.8:1	
HIV seropositive, n/N (%)	61/75 (81.3)	/
CD4 (cells/ul), median (IQR) (n = 70)	148 (74–312)	0.057
HIV+ (N = 61)	143 (70–282)	
HIV- (N = 8)	251 (149–767)	
HIVVL (cps/ml), median (IQR) (n = 56)	189 (20–118752)	/
Virological suppression^ψ^, n/N (%)	21/56 (37.5%)	/
ART exposure, n/N (%)	44/54 (81.5%)	/
Duration of ART (months), median (IQR)	15 (4–60)	
Stage IV disease, n/N (%)	47/62 (75.8)	
HIV+	39/49 (79.6)	0.27
HIV-	8/13 (61.5)	
IPI 4–5, n/N (%)	14/53 (26.4)	
HIV+	9/40 (22.5)	0.29
HIV-	5/13 (38.5)	
Performance status ≥2, n/N(%)	25/54 (46.3)	
HIV+	18/41 (43.9)	0.75
HIV-	7/13 (53.8)	
Cell of origin GC^α^, n/N (%)	39/52 (75)	
HIV+	32/41 (78.1)	0.68
HIV-	7/10 (70)	
MYC gene rearrangement, n/N (%)	7/52 (13.5)	
HIV+	6/39 (15.4)	0.67
HIV-	1/12 (8.3)	
Bone marrow involvement, n/N (%)	13/65 (20)	
HIV+	9/51 (17.6)	0.45
HIV-	4/14 (28.6)	
Ki-67 (%), median (IQR) (n = 71)	90 (80–95)	
HIV+	90 (80–95)	0.99
HIV-	90 (89–95)	
LDH (U/L), median (IQR) (n = 70)	722 (419–1288)	
HIV+ (n = 56)	749 (455–1299)	0.25
HIV- (n = 14)	591 (301–1293)	
Albumin (g/L), median (IQR) (n = 75)	33 (28–38)	
HIV+ (n = 60)	32 (27–38)	0.09
HIV- (n = 14)	35 (31–41)	
Β_2_microglobulin (mg/L), median (IQR) (n = 57)	4.7 (3.15–7.15)	
HIV+ (n = 46)	4.2 (2.7–7.8)	0.64
HIV- (n = 11)	4.8 (3.3–7.0)	
C-reactive protein (mg/L), median (IQR) (n = 62)	91 (47–146)	
HIV+ (n = 48)	117 (59–150)	0.017
HIV- (n = 13)	55 (16–94)	
Extranodal diseaseϷ, n/N (%)	54/68 (79.4)	
HIV+	40/56 (71.4)	0.16
HIV-	13/14 (92.9)	
Nodal disease, n/N (%)	69/74 (93.2)	
HIV+	56/60 (93.3)	0.58
HIV-	14/14 (100.0)	
Haemoglobin (g/dl), median (IQR) (n = 76)	10.5 (8.6–12.6)	
HIV+ (n = 61)	10 (8.6–12.5)	0.18
HIV- (n = 14)	11.6 (8.7–15.1)	
White cell count (x10^9^/L), median (IQR) (n = 76)	7.0 (5.0–10.5)	
HIV+ (n = 61)	7.1 (4.8–9.1)	0.66
HIV- (n = 14)	7.3 (4.9–13.3)	
Platelet count (x10^9^/L), median (IQR) (n = 76)	335 (242–513)	
HIV+ (n = 61)	336 (249–519)	0.17
HIV- (n = 14)	266 (128–489)	
Index chemotherapy protocol, n/N (%)		/
CHOP-based	44/56(78.6)	
R-CHOP based	8/56 (14.3)	
Other combination chemotherapy regimens	4/56 (7.2)	

HIVVL: HIV viral load, ART: antiretroviral therapy, GC: germinal center subtype, IPI: International prognostic index, CHOP: cyclophosphamide, doxorubicin, vincristine, and prednisone, R-CHOP: rituximab plus CHOP.

*p-values derived from analysis of HIV+ vs HIV-.

^ψ^: HIVVL<100cps/ml,

^α^: according to the Hans algorithm. The balance of the cases were of non-GC origin.

^Ϸ^ Extranodal disease includes cases with bone marrow, liver and spleen involvement.

**Table 2:** Dominant causes of death.

Cause of death	Number, n/N(%)
Confirmed or suspected sepsis	16/50 (32.0)
Renal failure	3/50 (6.0)
Progressive disease	4/50 (8.0)
Tumour lysis syndrome	2/50 (4.0)
Pneumonia	2/50 (4.0)
COVID-19	1/50 (2.0)
Tuberculosis	1/50 (2.0)
Unclear	10/50 (20.0)
Lost to follow-up after starting treatment	11/50 (22.0)

### Monocyte count, lymphocyte count and L:M results

Monocyte, lymphocyte and L:M ratios were similar between the people living with HIV and HIV negative individuals (Table 3). Lymphopenia (<1 x 10^9^/L) was present in 29/76 (38.2%) of the patients, and was not significantly more common in the people living with HIV ((26/61 (42.69%) vs 3/14 (21.4%) in the HIV negative group, p = 0.22). Monocytosis (>0.8 x 10^9^/L) was present in 18/76 (23.7%) of the patients, and did not differ in frequency according to HIV-status (p = 1). There was a significant positive correlation between the CD4 count and the lymphocyte count (r_s_ 0.56 (0.36–0.72); p<0.0001). On survival analysis, lymphopenia (<1.3 x 10^9^/L) and a CD4 count <150 cells/ul were both significantly associated with a shorter survival time in the people living with HIV (Table 4), while the monocyte count (>0.63 x 10^9^/L) was not. (Table 4).

**Table 3:** Immune cell-related data.

	All patients	People living with HIV	HIV negative	p-value*
Monocyte count (x 10^9^/L) [median (IQR)]	0.56 (0.43–0.79) (n = 76)	0.56 (0.43–0.79) (n = 61)	0.49 (0.42–0.78) (n = 14)	0.67
Lymphocyte count (x 10^9^/L) [median (IQR)]	1.17 (0.77–1.96) (n = 76)	1.14 (0.74–1.90) (n = 61)	1.47 (0.96–2.41) (n = 14)	0.20
Neutrophil count (x 10^9^/L) [median (IQR)]	4.59 (2.72–8.64) (n = 76)	4.96 (2.99–8.45) (n = 61)	3.33 (2.26–10.75) (n = 14)	0.19
L:M [median (IQR)]	2.12(1.39–3.11) (n = 75)	2.10 (1.35–2.79) (n = 61)	3.10 (1.31–5.50) (n = 13)	0.23
N:L [median (IQR)]	4.0 (2.09–8.08) (n = 76)	4.18 (2.46–8.22) (n = 61)	2.41 (1.29–9.90) (n = 14)	0.21
HLA-DR _ow_ monocytes (%) [median (IQR)]	26.2 (15.9–56.4) (n = 38)	24.1 (15.1–46.6) (n = 31)	36.0 (14.2–58.3) (n = 7)	0.68
Tregs (% of CD4 cells) [median (IQR)]	5.62 (1.65–9.27) (n = 29)	4.71 (1.62–9.23) (n = 23)	7.31 (3.43–12.39) (n = 6)	0.57
Tregs (Absolute count) [median (IQR)]	7.45 (2.33–17.8) (n = 29)	7.1 (2.07–16.37) (n = 23)	15.1 (5.41–44.1) (n = 6)	0.27
MO-Y [median (IQR)]	110.9 (102.7–115.8) (n = 57)	111.4 (103.9–115.6) (n = 46)	107.0 (98.5–115.8) (n = 10)	0.24
NE-SFL [median (IQR)]	47.8 (45.6–52) (n = 65)	45.7 (40.9–48) (n = 53)	44.2 (41.1–46.0) (n = 12)	0.25

*HIV+ vs HIV neg.

**Table 4:** Survival analysis.

	Median survival All Patients (months)	Median survival people living with HIV (months)
**Age**		
<60 years	3	3.5
≥60 years	4	3
Hazard ratio (CI)	0.93 (0.42–2.08)	0.9 (0.22–3.66)
p-value	0.87 (N = 73)	0.88 (N = 59)
**IPI**		
<4	6	5
≥4	0.73	0.7
Hazard ratio (CI)	4.65 (1.81–11.9)	5.92 (1.79–19.62)
p-value	0.001 (N = 53)	0.004 (N = 40)
**Stage**		
<4	24	24
4	4	3
Hazard ratio (CI)	0.45 (0.22–0.89)	0.45 (0.2–1.07)
p-value	0.02 (N = 60)	0.054 (N = 47)
**Extranodal sites**		
≥2	3.5	3.5
<2	9	6
Hazard ratio (CI)	1.98 (1.05–3.74)	1.66 (0.82–3.37)
p-value	0.035 (N = 64)	0.16 (N = 51)
**Performance status**		
≥2	1.75	1
<2	21	21
Hazard ratio (CI)	5.84 (2.77–12.31)	5.99 (2.52–14.35)
p-value	<0.0001 (N = 54)	<0.0001 (N = 41)
**CD4**		
<150 cells/ul	1.88	1.75
≥150 cells/ul	24	24
Hazard ratio (CI)	2.91 (1.56–5.42)	2.69 (1.42–5.11)
p-value	0.0008 (N = 67)	0.003 (N = 59)
**Virological suppression (<100 copies/ml)**		
Yes	6	6
No	2.4	2.4
Hazard ratio (CI)	0.52 (0.27–1.02)	0.52 (0.27–1.02)
p-value	0.06 (N = 55)	0.06 (N = 55)
**LDH**		
≥250 U/L	3	3
Normal range	16	14.8
Hazard ratio (CI)	1.4 (0.41–4.80)	1.26 (0.2–7.8)
p-value	0.06 (N = 68)	0.80 (N = 54)
**LDH**		
<1000 U/L	4	4.25
≥1000 U/L	3	1.75
Hazard ratio (CI)	0.55 (0.29–1.02)	0.49 (0.24–0.99)
p-value	0.06 (N = 68)	0.046 (N = 54)
**Β**_**2**_ **microglobulin**		
<2.5 mg/L	4	3.5
≥2.5 mg/L	3	3
Hazard ratio (CI)	0.69 (0.28–1.71)	1.15 (0.37–3.58)
p-value	0.42 (N = 55)	0.81 (N = 44)
**Monos**		
<0.63 x 10^9^/L	5	4.75
≥0.63 x 10^9^/L	1.75	1.75
Hazard ratio (CI)	0.57 (0.31–1.06)	0.69 (0.36–1.36)
p-value	0.07 (N = 73)	0.29 (N = 58)
**Neuts**		
≥8 x 10^9^/L	1.0	1.0
<8 x 10^9^/L	5.0	4.25
Hazard ratio (CI)	2.96 (1.39–6.32)	3.83 (1.4–10.4)
p-value	0.005 (N = 73)	0.008 (N = 59)
**L:M**		
<2:1	1.75	1.75
≥2:1	5	5
Hazard ratio (CI)	1.77 (0.97–3.23)	1.85 (0.96–3.56)
p-value	0.06 (N = 72)	0.07 (N = 58)
**N:L**		
≥ 6:1	1.4	1.0
< 6:1	5	5
Hazard ratio (CI)	2.95 (1.47–5.92)	2.94 (1.38–6.26)
p-value	0.002 (N = 72)	0.005 (N = 59)
**HLA-DR**_**low**_ **monocytes**		
<20%	18	24
≥20%	3	3
Hazard ratio (CI)	0.50 (0.21–1.16)	0.49 (0.19–1.25)
p-value	0.11 (N = 38)	0.14 (N = 31)
**Tregs (% of CD4)**		
≥5.17%	3	2
<5.17%	Not reached	Not reached
Hazard ratio (CI)	3.75 (1.39–10.1)	4.15 (1.35–12.8)
p-value	0.009 (N = 29)	0.01 (N = 23)
**Treg expression of CD39**		
≥93%	3	3
<93%	9	6
Hazard ratio (CI)	4.36 (1.14–16.62)	2.84 (0.8–10.1)
p-value	0.03 (N = 29)	0.11 (N = 23)
**MO-Y**		
<115.5	3	3
≥115.5	Not reached	Not reached
Hazard ratio (CI)	2.2 (1.06–4.55)	2.1 (0.96–4.78)
p-value	0.034 (N = 54)	0.06 (N = 43)
**NE-SFL**		
<50.5	3.5	3.5
≥50.5	1.75	1.88
Hazard ratio (CI)	0.52 (0.27–1.03)	0.611 (0.29–1.27)
p-value	0.06 (N = 63)	0.19 (N = 51)

LDH: Lactate Dehydrogenase, N/A: not applicable.

### Neutrophil count and N:L results

The neutrophil count and N:L ratios were similar between the people living with HIV and HIV negative individuals (Table 3). Neutropenia (<1.6 x 10^9^/L) was present in only 2/76 (2.6%) of the patients, both of whom were HIV negative. Neutrophilia (>7 x 10^9^/L) was present in 22/76 (28.9%), and did not differ in frequency according to HIV-status (p = 1). There was a significant positive correlation between the neutrophil count and the monocyte count (r_s_ 0.5 (0.31–0.66); p = <0.0001), but no significant relationship was seen between the neutrophil count and the lymphocyte count, the CD4 count, or the NE-SFL. On survival analysis, a neutrophil count above 8 x 10^9^/L and a N:L ratio ≥6:1 were both significantly associated with a shorter survival time in the people living with HIV (Table 4). No correlation was present between a neutrophil count above 8 x 10^9^/L and death due to sepsis.

### HLA-DR_low_ monocyte results

HLA-DR_low_ monocytes were assessed in 38 patients, 31 of whom (81.6%) were people living with HIV. The median HLA-DR_low_ monocyte count was 26.2%, and did not differ significantly according to HIV-status (Table 3). There was a significant positive correlation between the number of HLA-DR_low_ monocyte numbers and the absolute monocyte count (r_s_ 0.5 (0.2–0.71) p = 0.001) and the absolute neutrophil count (r_s_ 0.61 (0.36–0.78); p<0.0001). There was no significant correlation between the number of HLA-DR_low_ monocytes and the disease stage or the CD4 count. On survival analysis, survival times appeared substantially longer in patients with HLA-DR_low_ monocyte counts below 20%, but this finding lacked statistical significance, possibly due to the relatively limited number of cases with HLA-DR_low_ monocyte counts in this range (n = 11) (Table 4).

### Regulatory T-cell results

Treg numbers were assessed in 29 patients not yet on corticosteroids, with an HIV-seropositivity rate of 79.3%. The median Treg count as a proportion of CD4 T-cells was 5.62% (IQR 1.65–9.27), with a median absolute Treg count of 7.45 cells/ul (IQR 2.33–17.8). Treg counts did not differ significantly according to HIV-status (Table 3), but the proportion of CD4 cells made up by Tregs was significantly higher in the people living with HIV with CD4 counts <100 cells/ul (median 13.1% (IQR 8.96–26.14) vs 1.97% (IQR 0.96–4.71) in those with a CD4 count <100 and ≥100 cells/ul, respectively, p = 0.0009)). However, absolute Treg counts did not differ according to the CD4 count (7.81cells/ul (IQR 1.95–16.59) vs 5.2 cells/ul (IQR 2.07–16.37) in those with a CD4 count <100 and ≥100 cells/ul, respectively, p = 0.86)). Similarly, the expression of CD39 did not differ according to HIV-status, but was significantly higher in patients with low CD4 counts (median 93.8% (IQR 77.8–100) vs 53.0% (IQR 33.3–89.3%) in those with a CD4 count <100 and ≥100 cells/ul, respectively, p = 0.014)). Helios was expressed in a median of 28.4% of the Tregs (IQR 14–48%), while CD45RA (a marker expressed by naïve Tregs) was expressed on only 1.8% (IQR 0–9%). Neither Helios nor CD45RA expression on the Tregs differed according to HIV status.

There was a significant negative correlation between both the Treg count (as a proportion of CD4 cells; r_s_ -0.66 (-0.85 to -0.32) p = 0.0008) and CD39 expression (r_s_ -0.47 (-0.72 to -0.12) p = 0.01) with the CD4 count. On survival analysis in the global cohort, patients with a Treg count >5.17% (as a proportion of CD4 cells) and Treg expression of CD39>93% had significantly shorter survival (Table 4). Among patients with a Treg count of <5.17% of CD4 T-cells, 10/14 (71.4%) were alive at a median follow up time of 12 months. While the Treg% remained significantly associated with survival among the people living with HIV, the Treg expression of CD39 did not (possibly due in part to the more restricted sample size).

### MO-Y and NE-SFL results

The MO-Y was assessed in 57 patients at the time of enrollment, 46 of whom (80.7%) were people living with HIV. The median MO-Y value was 110.9, and did not differ according to HIV-status (Table 3). It showed no correlation to the CD4 or Treg counts, but MO-Y levels <115.5 were significantly associated with a poorer PS (r_s_ = 0.31(0.0007 to 0.56); p = 0.04). In addition, it showed marginally significant negative relationships to the monocyte count (r_s_ -0.25(-0.49 to 0.02); p = 0.06), the HLA-DR_low_ monocyte count (r_s_ = -0.24 (-0.5–0.05, p = 0.09) and the neutrophil count (r_s_ = -0.23 (-0.46–0.05, p = 0.09). The NE-SFL was measured in 65 patients and was similar between the people living with HIV and the HIV negative group. On survival analysis, survival times were significantly shorter in patients with MO-Y levels below 115.5, and were marginally longer in patients with a NE-SFL <50.5 (Table 4). Among patients with a MO-Y <115.5, 8/14 (57.1%) were alive at a median follow-up time of 26 months.

### Further survival analysis

In the global cohort, a high IPI, advanced disease stage, 2/more extranodal sites, the PS, a low CD4 count, virological suppression, lymphopenia, neutrophilia, a high N:L, a high Treg count, high Treg expression of CD39 and a low MO-Y were all associated with survival on univariate analysis (Table 4). Similar findings were seen among the people living with HIV, with the exception of an additional significant association seen in those with very high LDH levels (>1000 U/L), only marginally significant associations between survival and both the MO-Y levels and disease stage, and a lack of association with survival in relation to CD39 expression on the Tregs (likely due to the small sample size) (Table 4). For Cox proportional hazard analysis, too many variables showed a significant association with survival on univariate analysis to include in a single model owing to sample size constraints. Each immune-cell related variable significantly associated with survival was therefore included in a multivariate model with the IPI (as the most well recognized prognostic determinant) and the CD4 count (since several of the immune-cell variables with a significant association with survival showed correlations with the CD4 count). This demonstrated the CD4 count to be consistently associated with survival independently from the IPI (Tables 5–8 and 10). Both a neutrophil count >8 x 10^9^/L (Table 5) and a N:L >6:1 (Table 6) were significantly associated with survival independently from both the IPI and CD4 count, while the lymphocyte count (Table 7) and MO-Y (Table 8) were not. The number of cases with a Treg count was too low to include both the IPI and CD4 count in the multivariate model, and two separate analyses were therefore performed. Unfortunately, the Treg% did not meet the assumptions of proportionality, and could therefore not be incorporated into a multivariate model. However, Cox proportional hazard analysis of CD39 expression showed a marginally significant independent association between high level CD39 positivity and survival when adjusted for the IPI (Table 9), but no significant independent association when the CD4 count was included in the model (Table 10). The latter finding was duplicated among the people living with HIV, while multivariate analysis of CD39 expression and the IPI was not possible in this subgroup owing to the limited sample size.

**Table 5:** Cox proportional-hazards regression analysis for independent associations between the survival time and the IPI, CD4 count and a neutrophil count >8 x 10^9^/L.

	Coefficient	95% Confidence interval	p-value	Hazard ratio	95% Confidence interval
IPI ≥4					
Global cohort	0.63	-0.24 to 1.50	0.15	1.88	0.79 to 4.50
HIV+	0.84	-0.08 to 1.75	0.07	2.31	0.93 to 5.75
Neuts >8 x 10^9^/L					
Global cohort	0.93	0.12 to 1.73	0.03	2.52	1.13 to 5.65
HIV+	1.07	0.18 to 1.96	0.02	2.91	1.20 to 7.09
CD4 <150 cells/ul					
Global cohort	0.94	0.09 to 1.78	0.03	2.55	1.09 to 5.95
HIV+	0.98	0.10 to 1.85	0.03	2.66	1.10 to 6.39

**Table 6:** Cox proportional-hazards regression analysis for independent associations between the survival time and the IPI, CD4 count and a N:L >6:1.

	Coefficient	95% Confidence interval	p-value	Hazard ratio	95% Confidence interval
IPI ≥4					
Global cohort	0.48	-0.36 to 1.31	0.26	1.61	0.70 to 3.71
HIV+	0.67	-0.19 to 1.53	0.13	1.95	0.82 to 4.61
N:L >6:1					
Global cohort	0.77	0.04 to 1.51	0.04	2.17	1.04 to 4.54
HIV+	0.85	0.09 to 1.62	0.03	2.35	1.09 to 5.05
CD4 <150 cells/ul					
Global cohort	0.90	0.09 to 1.71	0.03	2.46	1.09 to 5.55
HIV+	0.80	-0.05 to 1.64	0.07	2.25	0.95 to 5.16

**Table 7:** Cox proportional-hazards regression analysis for independent associations between the survival time and the IPI, CD4 count and a lymphocyte count <1.3 x 10^9^/L.

	Coefficient	95% Confidence interval	p-value	Hazard ratio	95% Confidence interval
IPI ≥4					
Global cohort	0.49	-0.33 to 1.31	0.24	1.64	0.72 to 3.72
HIV+	0.67	-0.18 to 1.52	0.12	1.95	0.84 to 4.55
Lymphs <1.3 x 10^9^/L					
Global cohort	0.53	-0.34 to 1.39	0.24	1.69	0.71 to 4.03
HIV+	0.69	-0.23 to 1.60	0.14	1.99	0.80 to 4.98
CD4 <150 cells/ul					
Global cohort	0.86	0.07 to 1.66	0.03	2.37	1.07 to 5.27
HIV+	0.85	0.03 to 1.68	0.04	2.35	1.04 to 5.34

**Table 8:** Cox proportional-hazards regression analysis for independent associations between the survival time and the IPI, CD4 count and a MO-Y <115.5.

	Coefficient	95% Confidence interval	p-value	Hazard ratio	95% Confidence interval
IPI ≥4					
Global cohort	0.16	-0.91 to 1.22	0.77	1.17	0.41 to 3.38
HIV+	0.33	-0.74 to 1.41	0.54	1.40	0.47 to 4.10
MO-Y <115.5					
Global cohort	0.54	-0.60 to 1.67	0.36	1.71	0.55 to 3.33
HIV+	0.55	-0.59 to 1.69	0.35	1.73	0.55 to 5.41
CD4 <150 cells/ul					
Global cohort	1.13	0.15 to 2.12	0.02	3.11	1.16 to 8.33
HIV+	1.11	0.08 to 2.14	0.03	3.04	1.09 to 8.50

**Table 9:** Cox proportional-hazards regression analysis for independent associations between the survival time and the IPI and Treg expression of CD39.

	Coefficient	95% Confidence interval	p-value	Hazard ratio	95% Confidence interval
IPI ≥4					
Global cohort	0.66	-0.63 to 1.95	0.31	1.94	0.53 to 7.02
HIV+	Analysis not possible				
Treg CD39 ≥93%					
Global cohort	1.19	-0.14 to 2.52	0.08	3.29	0.87 to 12.42
HIV+	Analysis not possible				

**Table 10:** Cox proportional-hazards regression analysis for independent associations between the survival time and the CD4 count and Treg expression of CD39.

	Coefficient	95% Confidence interval	p-value	Hazard ratio	95% Confidence interval
CD4 <150 cells/ul					
Global cohort	1.76	0.52 to 3.0	0.006	5.79	1.67 to 20.0
HIV+	1.96	0.35 to 3.57	0.02	7.11	1.43 to 35.49
Treg CD39 ≥93%					
Global cohort	0.11	-1.01 to 1.24	0.84	1.12	0.36 to 3.45
HIV+	-0.06	-1.23 to 1.11	0.92	0.94	0.29 to 3.03

## Discussion

Immune dysfunction is an important contributor to the pathogenesis of DLBCL, and the immune signature of the tumour microenvironment (TME) has been shown to be a significant predictor of survival on gene expression profiling studies [15]. Peripheral blood monocyte, lymphocyte and neutrophil numbers are postulated to be a surrogate marker of the immune constituents of the TME, which may explain the well documented association between these cell numbers and survival. In this study, we have shown differential impacts of immune cell derangements among people living with HIV as compared to that reported in the international literature. For example, while a monocytosis is associated with poorer outcomes in studies from high income countries [3, 4, 6], this was not the case among the people living with HIV in this cohort, despite the fact that a monocytosis occurred with similar frequency in the people living with HIV as compared to the HIV-negative group. The lack of prognostic impact of monocytosis among the people living with HIV may relate to differences in the underlying cause of the monocyte proliferation (infective versus paraneoplastic) according to HIV-status, as CRP levels were significantly higher among the people living with HIV, suggesting the possibility of underlying infective comorbidities. Alternatively, the influence of the monocyte count may relate to limited exposure to Rituximab among the people living with HIV in this cohort. Rituximab therapy has historically not been used upfront in people living with HIV in our center owing to resource constraints. Monocytes have been reported to “shave” CD20/Rituximab complexes from the surface of malignant B-cells, thus reducing CD20 expression on tumour cells [16]. It is consequently possible that patients with higher monocyte counts have attenuated Rituximab efficacy, thus accounting for the poorer outcomes seen in the patients with monocytosis in high income countries.

In contrast to reports from other parts of the world [4, 7, 11], the L:M was not a strong prognostic indicator among people living with HIV in this cohort, showing only a marginally significant impact on survival. This effect was attributable to a negative prognostic impact associated with lymphopenia, which was shown on Cox proportional hazard analysis to be dependent on depression of the CD4 count in these patients. Lymphopenia per se was therefore not an independent predictor of survival among the people living with HIV. This finding contrasts with reports from other parts of the world, where lymphopenia is reportedly significantly independently associated with poorer outcomes in a dominantly HIV negative setting [17, 18].

As has been reported previously [19–21], we found the N:L (≥6:1) to be significantly associated with survival on univariate analysis in the people living with HIV. This has been postulated to be due the combined effects of a pro-inflammatory mileu (reflected by neutrophilia) and a degree of immunodeficiency (as evidenced by lymphopenia). This hypothesis is supported by the significant independent associations we found between both the presence of a neutrophilia (>8 x 10^9^/L) and a low CD4 count with survival on multivariate analysis. Interestingly, neutrophils were shown by Gregoire at al^3^ to support tumour cell growth and survival through production of BAFF/APRIL, and to induce changes in the stromal components of the TME which favour the support of malignant B-cells. At the same time, the stromal cells are induced to produce interleukin 8 and C-C motif ligand 2 (CCL2), thereby recruiting more neutrophils and monocytes to the TME, respectively. These dynamics within the TME may account for the neutrophilia and monocytosis seen in 28.9% and 23.7% of our patients, respectively, and may contribute to the poor survival rates seen in patients with a neutrophil count >8 x 10^9^/L.

Immunosuppressive (HLA-DR_low_) monocyte numbers were high (>25%) in this cohort, and did not differ significantly according to HIV-status. The findings are in agreement with those from a previous study performed in the USA,^11^ which showed an average of 35.2% HLA-DR_low_^11^ monocytes in patients with advanced stage DLBCL. However, in contrast to this report, we found no correlation between the number of HLA-DR_low_ monocytes and disease stage, and while survival rates appeared superior in patients living with HIV with HLA-DR_low_ mono counts <20%, this finding was not statistically significant. Notably, the HLA-DR_low_ monocyte count showed significant correlations with both the absolute monocyte and neutrophil counts, likely reflecting the effects of a sustained pro-inflammatory stimulus which drives both myeloid cell proliferation and induces homeostatic downregulation of the immune response. In addition, there was a marginally significant inverse correlation between the number of HLA-DR_low_ monocytes and monocyte fluorescence (MO-Y) levels, which were significantly associated with inferior survival on univariate survival analysis (possibly due to their relationship with a poorer PS). We have previously shown MO-Y levels to be correlated to monocyte CD64 expression, thus suggesting that this parameter reflects monocyte activation [13]. The negative association between low MO-Y levels and survival re-iterates the possibility of superior antitumour immunity in patients with robust monocyte activation. Interestingly, Lin et al showed evidence that the suppressive effect of HLA-DR_low_ monocytes was mediated by arginine metabolism, and that arginine supplementation partially reversed this phenotype [11]. The effect of arginine supplementation on MO-Y levels and, ultimately, disease outcomes, would be of interest in the setting of HIV-associated DLBCL (HIV-DLBCL). In addition, as the MO-Y is an easily measured, inexpensive and widely available parameter which is measured with the routine differential white cell count on several Sysmex haematology analysers, it holds appeal as a potential biomarker of antitumour immunity in the resource constrained setting. However, further validation of our findings with respect to this parameter is required.

Circulating Treg numbers are generally increased in individuals with cancer [22], a finding thought to contribute to immune tolerance to tumour cells and a cancer permissive mileu. Reports on peripheral blood Treg numbers in DLBCL have thus far proved conflicting, with a study from Taiwan reporting significantly higher Treg numbers in patients with DLBCL compared to healthy controls [23], and a second study from Poland reporting the opposite [24]. In our study, the median absolute Treg count was 7.45 cells/ul, making up a median of 5.62% of CD4 T-cells. Absolute Treg counts appeared substantially higher in the HIV-negative patients (15.1 cells/ul) as compared to the people living with HIV (7.1 cells/ul), but this finding lacked statistical significance, possibly due to the small number of HIV negative individuals included (n = 6). Unfortunately, a local reference interval for Tregs is not available, but a South African study assessing Treg numbers among people living with HIV which included 22 normal controls showed Tregs to comprise 1.3–7.5% of CD4 T-cells in healthy South Africans, with a median absolute Treg count of 30.47 cells/ul [25]. These levels are similar to those reported in the international literature, where Treg normal ranges of ~2.5–10% of CD4 cells [26, 27] and absolute counts of ~12.8–64.6 cells/ul [27, 28] have been reported. Based on these ranges, we conclude that the absolute number of Tregs is reduced in the setting of HIV-DLBCL, likely owing to the associated depression in overall CD4 T-cell counts. As has been previously described, we found the proportion of CD4 T-cells made up by Tregs to be significantly higher in patients with very low CD4 counts, with an accompanying increase in CD39 positive Tregs [29]. The latter reflect a subset of Tregs with particularly potent immunosuppressive function, as CD39 is an ectoenzyme which converts ATP to AMP, thereby releasing adenosine (which has T-cell suppressive function) [29]. While both Treg numbers (as a proportion of CD4 T-cells) and CD39 expression were associated with survival on univariate survival analysis, this is likely related to the close correlation between both of these factors and the CD4 count (as was confirmed for CD39 on multivariate analysis).

Survival rates in this study were very much poorer than is typically reported in high income countries, a finding which we have documented previously [30]. Important contributors to this finding were late case presentation and/or aggressive disease, as over 40% of the patients who died did so within one month of diagnosis, with 20% dying before any chemotherapy could be administered. The late presentation seen is likely at least partially due to delays in patient referral owing to an overburdened health care system, a problem which was exacerbated over the course of this study by the COVID-19 pandemic. Additional contributing factors likely include limitations in the available treatment modalities at CHBAH, along with other patient and disease-related factors. As mentioned above and as has been demonstrated previously [31–33], a CD4 count <150 cells/ul was independently associated with poorer survival in this study, a finding which was present in over half of the people living with HIV. Patients with CD4 counts in this range had been on ART for a significantly shorter duration, and were less likely to be virologically suppressed. These factors highlight the deleterious impact of poor HIV control at presentation in patients with HIV-DLBCL.

## Conclusion

The peripheral blood monocyte count and L:M are not strong prognostic indicators in HIV-DLBCL, and while a low lymphocyte count is associated with poorer survival, this is a product of low CD4 counts, and not lymphopenia per se. In contrast, a neutrophil count >8 x 10^9^/L has a significant independent negative prognostic impact in this setting, while evidence of neutrophil activation does not. Treg numbers are low in HIV-associated DLBCL, and do not have a significant bearing on survival independently from the CD4 count. Lastly, in contrast to previous reports in the dominantly HIV-negative context, the number of HLA-DR_low_ monocytes did not show a relationship with survival in this cohort, but monocyte activation (as evidenced by elevation of the MO-Y) did. We speculate that this finding could reflect a more robust antitumour immune response among individuals with elevation of the MO-Y. As an easily measured, inexpensive and widely available parameter, the MO-Y holds appeal as a potential biomarker of antitumour immunity. Further study in this regard would be of interest.

### Limitations

An important limitation to this study is the small number of patients who were HIV negative. As such, survival analysis of the HIV negative group was not possible. Additional limitations include the incomplete nature of the available data in a subset of the patients, and the limited exposure to Rituximab therapy in this cohort. Lastly, comparison of Treg numbers between the people living with HIV and the HIV-negative group was not adjusted for age and sex, which may have had an impact on these results.

## Supporting information

S1 Appendix (DOCX)

S2 Appendix (DOCX)

S3 Appendix (DOCX)

S1 Dataset (XLSX)
