# Correction: The prognostic impact of monocyte fluorescence, immunosuppressive monocytes and peripheral blood immune cell numbers in HIV-associated Diffuse Large B-cell Lymphoma

**DOI:** 10.1371/journal.pone.0331945

**Published:** 2025-09-11

**Authors:** Jenifer Vaughan, Moosa Patel, Denise Lawrie, Merriam Machaba, Tracey Wiggill

An error occurred in the coding of 6 of the patients who were lost to follow-up in the survival analysis. These patients were originally coded as having demised, where their outcome was in fact unknown.

This error has had a significant or marginally significant effect on the following results on survival analysis:

- The median survival time is 4 months (as opposed to the reported 3.5 months).
- The relationship between survival and the MO-Y parameter is only marginally significant (p-value 0.08 in the global cohort and 0.09 in the people living with HIV), as opposed to the statistically significant result originally reported in the global cohort (p value 0.03).
- There is a marginally significant association between survival and the HLA-DR_low_ monocyte count in the people living with HIV (p = 0.07), as opposed to the result lacking any statistical significance originally reported (p = 0.14).
- The monocyte count is significantly associated with survival in the global cohort (p = 0.04), but not in the people living with HIV (p = 0.11). The monocyte count was originally reported as being marginally significantly associated with survival in the global cohort (p = 0.07), but not in the people living with HIV (p = 0.29).
- HIV virological suppression is significantly associated with superior survival (p = 0.04). This association was originally reported as being marginally significant (p = 0.06).
- On Cox Proportional Hazards analysis, the neutrophil count is associated with survival independently from the IPI and CD4 count at a level of 6 x 10^9^/L as opposed to the reported 8 x 10^9^/L.

These changes have little effect on the conclusions of the paper, with the exception of a possible over-interpretation of the potential value of the MO-Y parameter.

The error also resulted in the following changes to the survival analysis which did not impact the statistical significance of the results:

- With respect to patient age, the p-value changed from 0.87 to 0.86 in the global cohort, but remained unchanged in the patients with HIV.
- With respect to the IPI score, the p-value changed from 0.001 to 0.0009 in the global cohort, but remained unchanged in the patients with HIV.
- With respect to disease stage, the p-value changed from 0.054 to 0.077 in the people living with HIV, and was unchanged in the global cohort.
- With respect to disease stage, the p-value changed from 0.054 to 0.077 in the people living with HIV, and was unchanged in the global cohort.
- With respect to the presence of extranodal disease the p-value changed from 0.035 to 0.011 in the global cohort, and from 0.16 to 0.2 in the patients with HIV.
- With respect to the CD4 count, the p-value changed from 0.0008 to 0.004 in the global cohort, and from 0.003 to 0.049 in the patients with HIV.
- With respect to the LDH level, the p-value changed from 0.06 to 0.25 in the global cohort, and from 0.8 to 0.64 in the patients with HIV.
- With respect to the Beta-2-microglobulin level, the p-value changed from 0.42 to 0.37 in the global cohort, and from 0.81 to 0.63 in the patients with HIV.
- With respect to the Neutrophil count, the p-value changed from 0.005 to 0.0002 in the global cohort, and from 0.008 to 0.039 in the patients with HIV.
- With respect to the L:M ratio, the p-value changed from 0.06 to 0.07 in the global cohort, and from 0.07 to 0.11 in the patients with HIV.
- With respect to the N:L ratio, the p-value changed from 0.002 to 0.003 in the global cohort, and from 0.005 to 0.013 in the patients with HIV.
- With respect to the NE-SFL, the p-value changed from 0.06 to 0.11 in the global cohort, and from 0.19 to 0.279 in the patients with HIV.

This has also resulted in errors in Tables 4, 5 and 6. Please see the correct Tables here.

**Table 4:** Survival analysis.

	Median survival All Patients (months)	Median survival people living with HIV (months)
Age		
< 60 years	3	3.5
≥ 60 years	4	3
Hazard ratio (CI)	0.93 (0.42-2.08)	0.9 (0.22-3.66)
p-value	0.86	0.88
	(N = 73)	(N = 59)
IPI		
< 4	6	5
≥ 4	0.73	0.7
Hazard ratio (CI)	4.65 (1.81-11.9)	5.92 (1.79-19.62)
p-value	0.0009	0.004
	(N = 53)	(N = 40)
Stage		
< 4	24	24
4	4	3
Hazard ratio (CI)	0.45 (0.22-0.89)	0.45 (0.2-1.07)
p-value	0.02	0.077
	(N = 60)	(N = 47)
Extranodal sites		
≥ 2	3.5	3.5
< 2	9	6
Hazard ratio (CI)	1.98 (1.05-3.74)	1.66 (0.82-3.37)
p-value	0.011	0.2
	(N = 64)	(N = 51)
Performance status		
≥ 2	1.75	1
< 2	21	21
Hazard ratio (CI)	5.84 (2.77-12.31)	5.99 (2.52-14.35)
p-value	<0.0001	<0.0001
	(N = 54)	(N = 41)
CD4		
< 150 cells/ul	1.88	1.75
≥ 150 cells/ul	24	24
Hazard ratio (CI)	2.91 (1.56-5.42)	2.69 (1.42-5.11)
p-value	0.004	0.049
	(N = 67)	(N = 59)
Virological suppression (<100 copies/ml)		
Yes	6	6
No	2.4	2.4
Hazard ratio (CI)	0.52 (0.27-1.02)	0.52 (0.27-1.02)
p-value	0.04	0.04
	(N = 55)	(N = 55)
LDH		
≥ 250 U/L	3	3
Normal range	16	14.8
Hazard ratio (CI)	1.4 (0.41-4.80)	1.26 (0.2-7.8)
p-value	0.25	0.64
	(N = 68)	(N = 54)
LDH		
< 1000 U/L	4	4.25
≥ 1000 U/L	3	1.75
Hazard ratio (CI)	0.55 (0.29-1.02)	0.49 (0.24-0.99)
p-value	0.06	0.046
	(N = 68)	(N = 54)
Β_2_microglobulin		
< 2.5 mg/L	4	3.5
≥ 2.5 mg/L	3	3
Hazard ratio (CI)	0.69 (0.28-1.71)	1.15 (0.37-3.58)
p-value	0.37	0.63
	(N = 55)	(N = 44)
Monos		
< 0.63 x 10^9^/L	5	4.75
≥ 0.63 x 10^9^/L	1.75	1.75
Hazard ratio (CI)	0.57 (0.31-1.06)	0.69 (0.36-1.36)
p-value	0.04	0.11
	(N = 73)	(N = 58)
Neuts		
≥ 6 x 10^9^/L	1.0	1.0
< 6 x 10^9^/L	5.0	4.25
Hazard ratio (CI)	2.96 (1.39-6.32)	3.83 (1.4-10.4)
p-value	0.0002	0.039
	(N = 73)	(N = 59)
L:M		
< 2:1	1.75	1.75
≥ 2:1	5	5
Hazard ratio (CI)	1.77 (0.97-3.23)	1.85 (0.96-3.56)
p-value	0.07	0.11
	(N = 72)	(N = 58)
N:L		
≥ 6:1	1.4	1.0
< 6:1	5	5
Hazard ratio (CI)	2.95 (1.47-5.92)	2.94 (1.38-6.26)
p-value	0.005	0.013
	(N = 72)	(N = 59)
HLA-DR_low_ monocytes		
< 20%	18	24
≥ 20%	3	3
Hazard ratio (CI)	0.50 (0.21-1.16)	0.49 (0.19-1.25)
p-value	0.11	0.07
	(N = 38)	(N = 31)
Tregs (% of CD4)		
≥ 5.17%	3	2
< 5.17%	Not reached	Not reached
Hazard ratio (CI)	3.75 (1.39-10.1)	4.15 (1.35-12.8)
p-value	0.009	0.01
	(N = 29)	(N = 23)
Treg expression of CD39		
≥ 93%	3	3
< 93%	9	6
Hazard ratio (CI)	4.36 (1.14-16.62)	2.84 (0.8-10.1)
p-value	0.03	0.11
	(N = 29)	(N = 23)
MO-Y		
< 115.5	3	3
≥ 115.5	Not reached	Not reached
Hazard ratio (CI)	2.2 (1.06-4.55)	2.1 (0.96-4.78)
p-value	0.08	0.09
	(N = 54)	(N = 43)
NE-SFL		
< 50.5	3.5	3.5
≥ 50.5	1.75	1.88
Hazard ratio (CI)	0.52 (0.27-1.03)	0.611 (0.29-1.27)
p-value	0.11	0.279
	(N = 63)	(N = 51)

LDH: Lactate Dehydrogenase, N/A: not applicable.

**Table 5:** Cox proportional-hazards regression analysis for independent associations between the survival time and the IPI, CD4 count and a neutrophil count >8 x 10^9^/L.

	Coefficient	95% Confidence interval	p-value	Hazard ratio	95% Confidence interval
IPI ≥ 4					
Global cohort	0.63	−0.24 to 1.50	0.15	1.88	0.79 to 4.50
HIV+	0.84	−0.08 to 1.75	0.07	2.31	0.93 to 5.75
Neuts >6 x 10^9^/L					
Global cohort	0.93	0.12 to 1.73	0.03	2.52	1.13 to 5.65
HIV+	1.07	0.18 to 1.96	0.02	2.91	1.20 to 7.09
CD4 < 150 cells/ul					
Global cohort	0.94	0.09 to 1.78	0.03	2.55	1.09 to 5.95
HIV+	0.98	0.10 to 1.85	0.03	2.66	1.10 to 6.39

**Table 6:** Cox proportional-hazards regression analysis for independent associations between the survival time and the IPI, CD4 count and a N:L > 6:1.

	Coefficient	95% Confidence interval	p-value	Hazard ratio	95% Confidence interval
IPI ≥ 4					
Global cohort	0.41	−0.50 to 1.33	0.38	1.51	0.60 to 3.99
HIV+	0.81	−0.12 to 1.74	0.09	2.24	0.88 to 5.70
N:L > 6:1					
Global cohort	1.12	0.25 to 1.99	0.01	3.06	1.28 to 7.30
HIV+	1.07	0.17 to 1.98	0.032	2.93	1.18 to 7.22
CD4 < 150 cells/ul					
Global cohort	1.08	0.12 to 2.04	0.03	2.93	1.12 to 7.66
HIV+	0.95	0.01 to 1.90	0.049	2.59	1.01 to 6.69

A member of the Editorial Board reviewed the corrected findings and advised that they support the results and conclusions reported in the article.
