# Supplementary material for: The prognostic impact of monocyte fluorescence, immunosuppressive monocytes and peripheral blood immune cell numbers in HIV-associated Diffuse Large B-cell Lymphoma
Source: PLoS One. 2023 Jan 11;18(1):e0280044. doi: 10.1371/journal.pone.0280044 (PMC9833596; doi:10.1371/journal.pone.0280044)
Supplement: S1 Appendix — (DOCX) [file pone.0280044.s001.docx]

Supplementary Figure 1

Patients were included in the study according to the adequacy of their diagnostic biopsy sample (a pre-requisite for the original study entitled “Evaluation of the association between tumour enrichment with M2-macrophages and survival among South African patients with Diffuse Large B-cell Lymphoma”). The inclusion of patients for further blood testing was complex for several reasons:

- Access to inpatients was restricted during part of this study due to COVID-19 related measures, so that blood could not be collected specifically for research purposes. As such, the testing was dependent on blood samples submitted for routine diagnostic tests.
- In some instances, corticosteroids, radiation or chemotherapy were commenced before referral or before the research tests samples could be collected, in which case further testing was omitted (unless the diagnostic biopsy was performed while already on steroids). Some of the research test results (specifically the Treg numbers and HLA-DR_low_ monocyte numbers) were also excluded when the history of corticosteroid exposure was unclear.
- Some of the testing was unsuccessful due to random technical errors. In addition, the Treg methodology was found to be unsuccessful part way through the study, and results analysed with this original method were consequently excluded.
- Many of the routine tests assessed (such as LDH, B2-microglobulin, CD4, HIVVL, etc) were not requested by the attending clinicians.

Approximately 100 patients were referred to the CHBAH with DLBCL over the study period

Approximately 24 patients did not have an adequate biopsy for inclusion in the parent study

Research-specific tests (flow cytometry for Tregs and HLA-DR low monocytes) omitted or results excluded

HLA-DR test results difficult to interpret due to granulocyte activation in 3 patients.

Appropriate tubes unavailable in 6 patients

Corticosteroid exposure was unclear at the time of testing in 6 patients

21 patients commenced on corticosteroids, chemotherapy or radiation before testing was possible.

76 patients had an adequate biopsy

MO-Y and NE-SFL testing failed in 2 patients, was excluded in 3 patients due to the presence of circulating tumour cells, was not possible due to tube unavailability in 6 patients, and was erroneously omitted in 8 patients at the time of enrollment.

Treg test methodology found to be unsatisfactory part way through the study: results excluded in 12 patients

Excluded
