# Supplementary material for: The prognostic impact of monocyte fluorescence, immunosuppressive monocytes and peripheral blood immune cell numbers in HIV-associated Diffuse Large B-cell Lymphoma
Source: PLoS One. 2023 Jan 11;18(1):e0280044. doi: 10.1371/journal.pone.0280044 (PMC9833596; doi:10.1371/journal.pone.0280044)
Supplement: S2 Appendix — (DOCX) [file pone.0280044.s002.docx]

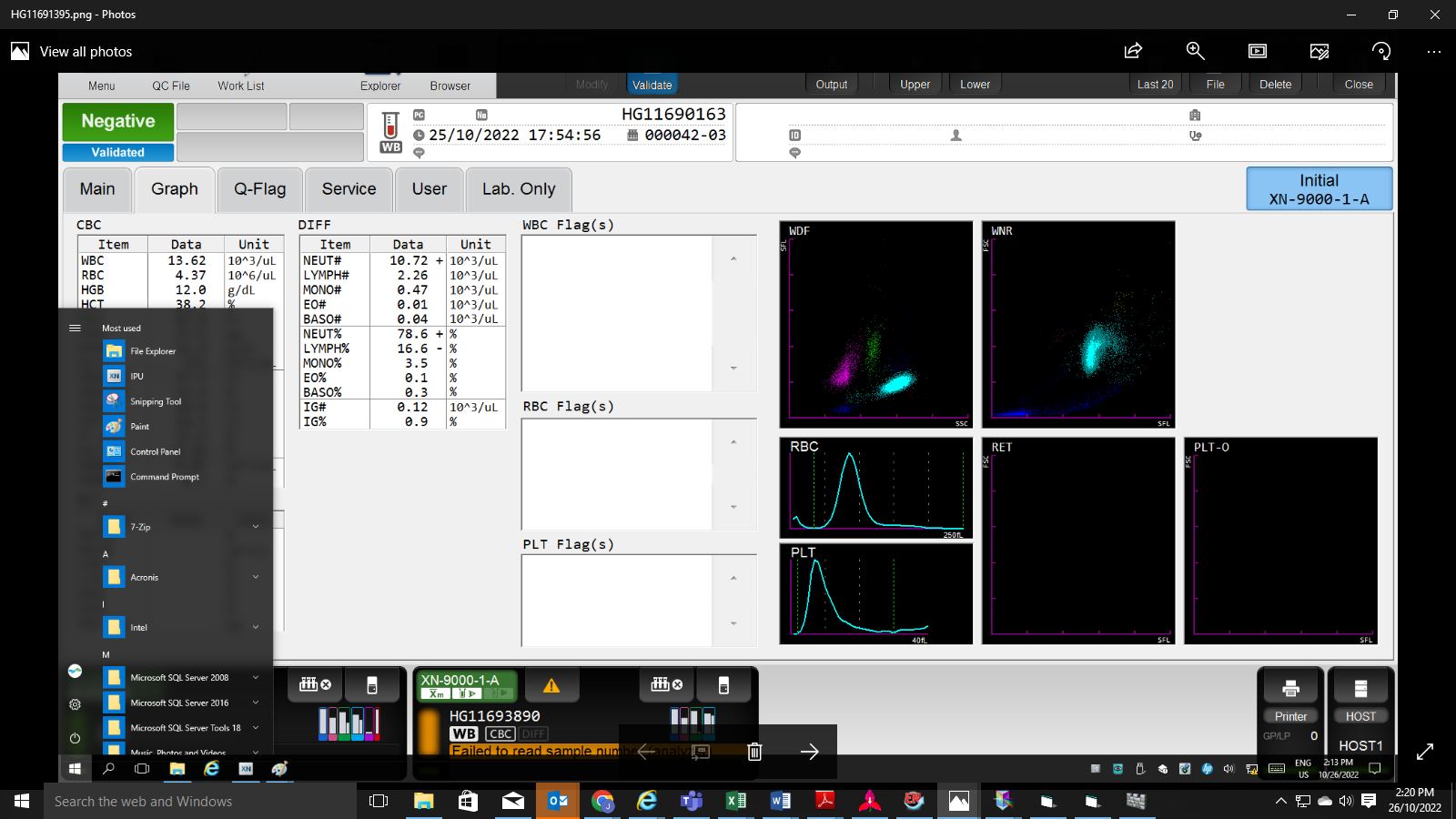

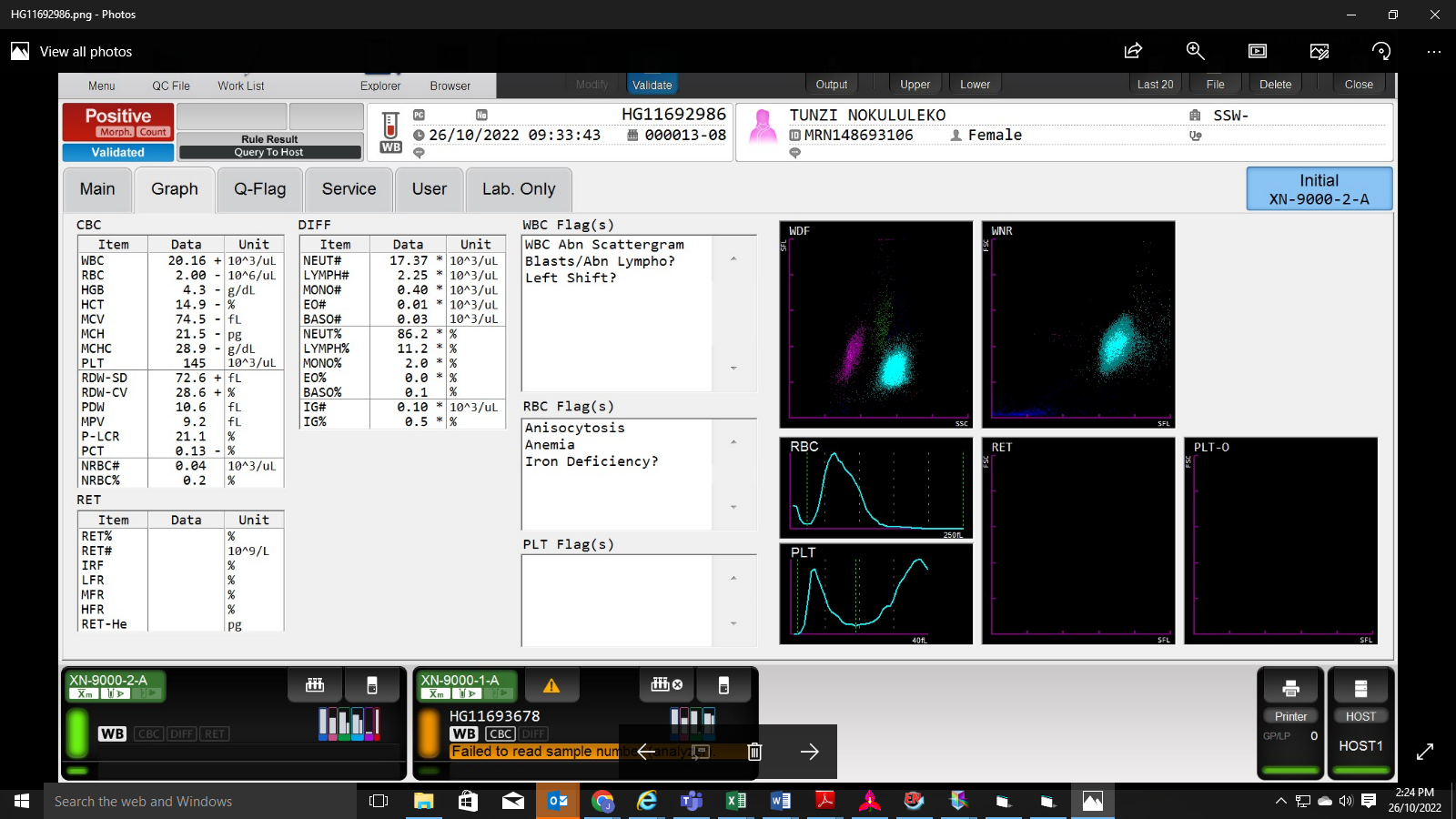


B

A
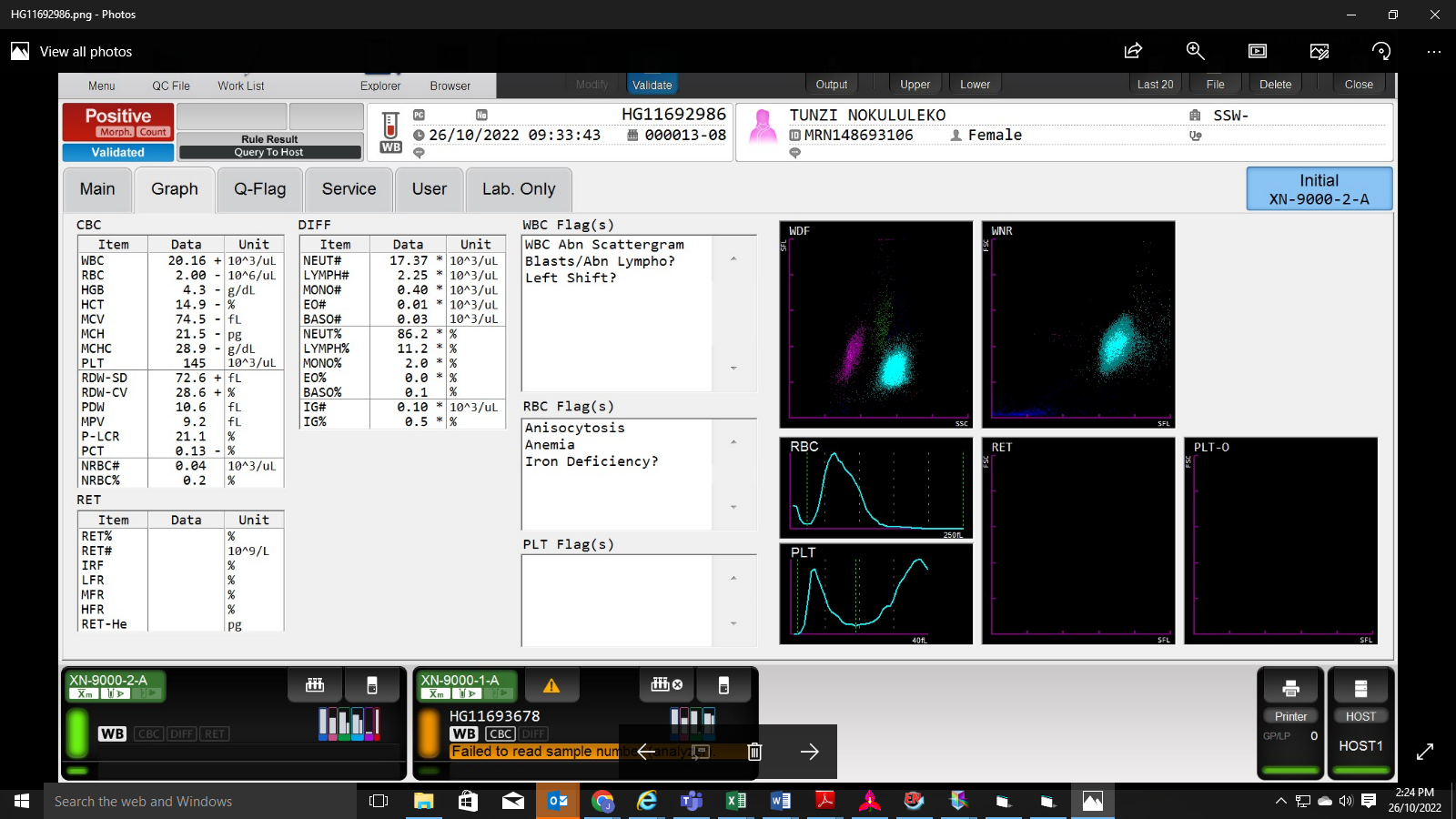


Supplementary Figure 2: MO-Y and NE-SFL are derived from the centroid of the SFL (Y axis) signal of the monocyte and neutrophil populations, respectively in the WDF scattergram. In the above scatterplots, the turquoise populations are the neutrophils, the magenta populations are the lymphocytes and the green populations are the monocytes. Case A has a NE-SFL of 40.4, and a MO-Y of 79.5. Case B has a NE-SFL of 73.1 and a MO-Y of 125.6.
