# Supplementary material for: The prognostic impact of monocyte fluorescence, immunosuppressive monocytes and peripheral blood immune cell numbers in HIV-associated Diffuse Large B-cell Lymphoma
Source: PLoS One. 2023 Jan 11;18(1):e0280044. doi: 10.1371/journal.pone.0280044 (PMC9833596; doi:10.1371/journal.pone.0280044)
Supplement: S3 Appendix — (DOCX) [file pone.0280044.s003.docx]

**HLA-DR_low_ monocyte sample preparation**

A 0.5ml aliquot of peripheral blood was washed three times with 14ml of commercially sourced phosphate buffered saline (PBS) at pH 7.3±2 (Oxoid LTD, Basingstoke, UK). Post washing, the sample was reconstituted to 0.5ml with PBS and the white cell count determined. Cells were then diluted to achieve the recommended cellular concentration (±10 000 cells/µl). 100µl of this cell concentrate, containing ± 10 000cells/ µl, was placed in a 5ml tube and incubated with 10µl of Anti-CD14 fluorescein isothiocyanate (FITC) (BD Biosciences, San Jose, CA, USA), 5µl of Anti- CD45 phycoerythrin (PE) (Beckman Coulter Inc, Brea, USA) and 10µl of Anti- HLA-DR- pacific blue (PB) (Beckman Coulter/Immunotech SAS, Marseille, France) for 15 minutes in the dark at 22-25°C. Post incubation 3ml of Isotonic Ammonium Chloride pH 7.1-7.4 (8.99%NH4Cl, 0.84% NaHCO3 and 0.0372%EDTA, Merck, Darmstad, Germany was added to the tube for 10 minutes at room temperature to lyse the red blood cells. Post lysing the samples were spun at 3000g for 3 minutes, decanted, washed once with PBS and reconstituted to 0.5ml with PBS. Flow cytometer acquisition and analysis was performed on a minimum of 50 000 leukocytes on a Beckman Navios flow cytometer. Although no spill over or increased background signal was envisaged with this panel of monoclonal antibodies, fluorescence minus one (FMO) controls were prepared to validate the region positions and cut offs for the positive and negative populations. Monocytes were gated according to light scatter and CD14 expression, and the proportion with low or absent HLA-DR expression reported as a percentage thereof (Supplementary Figure 1). The threshold for negative expression was set at the upper limit of granulocyte expression of HLA-DR (as these cells typically do not express HLA-DR unless activated). Patients who showed some expression of HLA-DR on their granulocytes were excluded, as were those who had been commenced on corticosteroids.

**Supplementary Figure 1: HLA-DR_low_ monocyte anaysis**

Monocytes (red events) and granulocytes (purple events) were gated by virtue of their expression of CD14, CD45 and light scatter, respectively.

In a CD14 (FITC)/HLA-DR (PE) plot, the upper limit of HLA-DR expression on the granulocytes (as the internal negative control) was set using a quadrant overlay.

After gating on the monocytes, the proportion with HLA-DR expression below that of the upper limit seen in the granulocytes was determined. The above case showed low HLA-DR expression on 14.07% of the monocytes.

In contrast, the example below showed low expression of HLA-DR in 83.7% of the monocytes.

**Regulatory T-cell (Treg) sample preparation**

50 µl of whole blood was incubated in a DuraClone IM Treg tube (Tube 1) (Beckman Coulter Inc, Brea, USA)) for 15 minutes in the dark. Thereafter, 3 ml of PBS were added to the tube, and it was centrifuged at 500g for 5 minutes at room temperature. The supernatant was then aspirated and the cell pellet resuspended in 50µl of 100% fetal calf serum and incubated with 5µl of PerFix nc Buffer 1 (fixative reagent) for 15 minutes in the dark. 400 µl of PerFix Buffer 2 (permeabilising reagent) was then added to Tube 1, and the contents transferred to the DuraClone IM Treg Tube 2. This was then incubated in the dark for 1 hour. After adding 3ml of PBS to Tube 2, it was incubated for 5 minutes and centrifuged at 500g for 5 minutes. The supernatant was then aspirated, and the cell pellet resuspended with 3 ml of PerFix Buffer 3. The tube was then centrifuged again at 500g for 5 minutes, the supernatant aspirated, and the pellet resuspended in 500µl of PerFix Buffer 3. Flow cytometer acquisition and analysis was performed on a minimum of 50 000 leukocytes on a Beckman Navios flow cytometer. Lymphocytes were gated according to light scatter and CD45 expression. Of the lymphocytes, the C3+CD4+ cells were then gated, followed by gating of the CD25+CD4+ T-cells, the FOXP3+ CD4+CD25+ T-cells, the Helios+ FOXP3+CD4+CD25+ T-cells, the CD39+ FOXP3+CD4+CD25+ T-cells and the CD45Ra+FOXP3+ CD4+CD25+ T-cells. For the latter gates, the expression threshold for negative expression was set at the level of the background CD3+CD8+CD4- lymphocytes. Patients who had been commenced on corticosteroids were excluded.

**Supplementary Figure 2: Treg analysis**
